# A systematic review of performance-based functional capacity measures for use in Huntington’s disease and evaluation of their suitability for clinical trials

**DOI:** 10.1177/18796397251330846

**Published:** 2025-04-03

**Authors:** Tayela M Prichard, Cali M Roiboit, Meg E Rankin, Yifat Glikmann-Johnston, Mark F Gordon, Julie C Stout

**Affiliations:** 1 Turner Institute for Brain and Mental Health, School of Psychological Sciences, Monash University, Clayton, VIC, Australia; 2 Teva Pharmaceuticals, West Chester, PA, USA

**Keywords:** Huntington's disease, performance-based measure, functional capacity, activities of daily living

## Abstract

**Background:**

Huntington's disease (HD) leads to a decline in functional capacity, affecting daily life tasks. Assessing functional capacity in clinical trials is crucial to evaluate treatment effectiveness and substantiate the clinical meaningfulness of more sensitive and reliable measures. Clinician rating scales are commonly used, but performance-based measures of functional capacity may offer advantages, however, there is no consensus on the suitability of existing performance-based measures for use in HD.

**Objective:**

We applied a Consensus-based Standards for the selection of health Measurement INstruments (COSMIN) approach to evaluate the potential suitability of performance-based functional capacity measures for HD clinical trials. We also used criteria developed with expert input to assess these measures.

**Methods:**

We conducted a systematic search of relevant databases and screened 1924 articles for inclusion criteria.

**Results:**

We included a total of 89 articles on 33 performance-based functional capacity measures. Measures were rated from Very Low to Moderate suitability for use in HD clinical trials. DriveSafe DriveAware and EcoKitchen were the only measures tested in HD participants and were rated as having Moderate and Very Low suitability respectively, highlighting the need for further evaluation. Additionally, the Brief University of California San Diego Performance-based Skills Assessment (UCSD UPSA-B) and the Virtual Reality Functional Capacity Assessment Tool (VRFCAT), were identified as potentially useful, also rated Moderate.

**Conclusions:**

Multiple performance-based functional capacity measures show potential for use in patients with HD, pending further investigation.

## Introduction

Overt signs and symptoms of Huntington's disease (HD), including cognitive, motor and neuropsychiatric features, typically manifest in middle-age.^1^ In combination, these signs and symptoms limit a person's ability to perform tasks in daily life and work, with such limitations commonly being assessed using ‘functional capacity’ measures. In clinical trials, functional capacity is typically assessed using either self-report or clinician-rated measures. Alternatively, functional capacity can be measured using performance-based measures, which quantify a person's ability to perform everyday functional tasks under standardized conditions. Functional capacity measures have important roles for detecting treatment benefits in clinical trials, because regulatory approval requires evidence of a meaningful effect “on how a patient feels, functions, or survives”.^2^ Current practice for assessing functional capacity has favored the use of rating scales rather than performance-based measures; however, performance-based measures have potential advantages in that they are a direct method of establishing actual functional abilities, limiting the rater biases inherent in observer and self-report measures.^3^ The goal of this review was to evaluate the literature on performance-based measures of everyday functional activities to ascertain the potential of such tools to be adopted for HD research and clinical trials.

The functional rating scale most commonly used in HD clinical trials is the Unified Huntington's Disease Rating Scale (UHDRS) Total Functional Capacity (TFC) scale.^4^ The UHDRS TFC scale is a five-item measure that involves a clinician asking people about their ability to perform daily tasks on occupation, finances, domestic chores, activities of daily living, and the care level required to obtain an overall rating.^4^ Other common measures include the Clinical Global Impressions (CGI) scale and two additional clinician-rated functional capacity scales included as part of the UHDRS, the Functional Assessment Scale (FAS) and the Independence Scale (IS).^4,5^ Of these, the TFC is the most frequently used in clinical trials. Despite their frequent use, the UHDRS TFC and other functional capacity rating scales have important limitations that may be overcome in performance-based assessments of function. Clinician-rated measures rely on information about function from limited periods of clinical observation and occur in the artificial setting of the clinic; as such, they are unable to rate function in the wider context of day-to-day life. Interestingly, outcomes from clinician versus patient self-ratings of functional capacity in HD yield different impressions, with clinicians generally rating patients as higher functioning than participants do themselves.^6^ Although patient-focused drug development approaches elevate the voice of the patient in describing their own experiences over those of observers,^7^ loss of insight in HD can yield overestimates of self-reported abilities in all areas, including function.^8–10^ Rating scales that use observer reports from close others are seldom used in HD clinical trials and are subject to their own limitations, including rater biases, memory lapses, and the stress levels of the rater.^6,11,12^ Conversely, performance-based measures of function directly evaluate a person's ability to complete various tasks relevant to function through simulated activities. As such, they have the potential to more accurately and objectively assess functional abilities in HD and may mitigate the limitations of clinician, observer, and self-ratings.^11–14^

Although sensitive and reliable measures of everyday function are essential for characterizing the impact of the cognitive, motor and neuropsychiatric manifestations of HD, measures such as the UHDRS TFC and IS are not always fit for purpose. These measures have been repeatedly shown to be insensitive to more subtle signs of HD progression, which they were not designed to detect.^15,16^ The TFC demonstrates a very limited dynamic range with scores ranging from only 0–13, with 13 indicating unimpaired function. In practice, this range is further truncated due to most studies and trials including people with scores of at least 6 or more, because those with lower scores are too far declined in function to undertake many of the study and trial assessments.^17,18^ Furthermore, in the earliest stage of HD, and in virtually all premanifest people with HD, the perfect score of 13 is exceedingly common, and as such there is no potential to capture improvement which may occur in the context of an effective treatment. Indeed, previous reviews have been unable to recommend existing rating scales of function used in HD for screening or to assess disease severity due to ceiling effects in early stages of HD and the limited evaluation of their clinimetric properties.^19^ Despite the various limitations of the UHDRS functional capacity measures, they continue to be widely used in HD clinical trials due to an absence of more appropriate measures. New patient-reported measures are in development to address this limitation for future clinical trials, including the Functional Rating Scale 2.0 (FuRST 2.0)^20^ and the Huntington's Disease Everyday Functioning (Hi-DEF) Scale^21^; however, performance-based measures of function have the potential further benefit of high ecological and face validity, meaning results may be more closely linked to everyday function.^22^

In addition to evaluating the efficacy of medical interventions in clinical trials, another role for measures of functional capacity in clinical trials is to support the clinical meaningfulness of other, more sensitive endpoints, such as cognitive measures. Although cognitive changes often underly impairment in everyday activities, cognitive measures themselves frequently lack face validity for establishing functional capacity on their own, despite their sensitivity.^23^ To illustrate this further, the Symbol Digit Modalities Test (SDMT) is a highly sensitive and frequently used cognitive measure in HD clinical trials, research, and neuropsychological evaluations.^24^ The SDMT is a speeded paper-based test requiring a person to refer to a key at the top of the page showing a series of symbols paired with numbers and fill in the missing numbers across a series of rows showing only the symbols. Despite the sensitivity of the SDMT in HD and other conditions, it lacks face validity in that it does not mimic any everyday functional tasks. Associations between SDMT performance and measures of function can be used to support the case that the SDMT is relevant to everyday function.^25^ In the context of clinical trials where sensitivity and the demonstration of functional relevance of outcomes is essential, the combination of measures can present the best scenario for both detecting treatment effects and showing that these effects are clinically meaningful.

Cognitive test batteries designed for HD clinical trials, such as the Huntington's Disease Cognitive Assessment Battery (HD-CAB), and the UHDRS cognitive function subscales, are highly sensitive to disease progression.^4,26,27^ Importantly, well-chosen cognitive measures can typically detect cognitive impairment before clinically apparent functional decline occurs; therefore, they may serve a particularly important role in prodromal HD clinical trials.^28^ Despite the sensitivity and reliability of cognitive measures, how changes in these measures eventually translate to everyday function is understudied and thus the clinical meaningfulness of change based solely on cognitive measures cannot be assumed. Strong associations between sensitive and reliable cognitive measures and measures of everyday functional capacity have the potential to reveal implications for the clinical meaningfulness of cognitive and other more sensitive symptom measures. How cognitive measures and performance-based measures of functional capacity relate in HD, however, has been the subject of only limited research.^14,29^

Previous reviews have yielded recommendations and suggestions for performance-based measures of physical function in HD, such as those designed to assess gait and balance, and rating scales assessing everyday functional capacity, which is a broader construct that assesses everyday activities^19^; however, no research has systematically evaluated *performance-based measures* of functional capacity. Measures that more broadly assess every day, higher-level functional skills (e.g., financial management and cooking) may have greater utility in HD clinical trials because they better reflect the range of cognitive skills that enable people to function independently, which declines gradually as people with HD progress.^14^ Due to the potential utility of these types of measures, we examined published performance-based functional capacity measures which have been developed for or used in various relevant populations to determine their potential suitability for use in HD clinical trials. A suitable performance-based functional capacity measure may help establish the effect of a treatment on function, supporting regulatory approval, as well as provide support for the clinical meaningfulness of cognitive measures that may have greater sensitivity to the subtle clinical features of HD. We applied a Consensus-based Standards for the selection of health Measurement Instruments (COSMIN) approach to evaluate the potential suitability of performance-based functional capacity measures for use in HD clinical trials. As such, we also evaluated measures for their suitability to be used as an adjunct for evaluating clinical meaningfulness of cognitive measures for HD clinical trials.

## Methods

We conducted a literature search of the databases Ovid Medline (1946 to April 2024), PsycINFO via Ovid (1806 to April 2024), Embase via Ovid (1974 to April 2024), and Web of Science Core Collection (1900 to April 2024). Table 1 shows the Medical Subject Headings (MeSH) and text-words for database Ovid Medline that we developed by creating a concept map of key words and populations found in the literature. The search terms differed by database due to differences in the usage and categorization of MeSH (see Supplemental Material Appendix A, Table A1, A2 and A3). We then entered these words into the database to find relevant subject headings that captured a broad range of words and topics for the same concept. Words that were not captured by subject headings were entered as text-words. The search results were limited to English language. We conducted the literature search in the following two stages.

**Table 1.** Literature search strategy ovid medline MeSH terms and text-words.

MeSH	activities of daily living/ AND psychological tests/ OR neuropsychological tests/ AND Alzheimer disease/ OR exp Huntington Disease/ OR Parkinson disease/ OR *Cognitive Dysfunction / di [Diagnosis] AND exp Psychometrics/
Key Words	function* OR activit* daily li*AND measure OR assessment OR test OR tool AND Huntington* OR Westphal variant OR Alzheimer* OR Parkinson* AND validity AND performance

In Stage 1 study titles and abstracts were screened by TP and CR for relevant measures with the following inclusion and exclusion criteria. We aimed to identify measures that could be used to establish effects on everyday function in clinical trials for HD and to support the clinical meaningfulness of effects on cognitive measures. As such, our inclusion and exclusion criteria were informed by previous research identifying desirable characteristics for outcome measures in clinical trials for HD.^30,31^ Inclusion criteria:

- Study available in English language.
- Psychometric or ecological validity study or systematic review.
- Cognitive and motor disorders including Huntington's, Alzheimer's, Parkinson's diseases, and multiple sclerosis, psychiatric conditions such as schizophrenia, or older adult participants. We chose to include measures tested in non-HD populations in our review as very few performance-based measures of everyday function have been evaluated in HD populations.
- Measures underlying aspects of cognition, such as executive function.
- Measures instrumental/complex activities of daily living and/or real-world function, job performance and/or functional capacity.

Exclusion criteria:

- Designed to be over 25 min in length as this would be undesirable in a clinical trial context.^31^
- Tasks with primary focus on motor function, such as balance or walking abilities, which have limited implications for the range of cognitive abilities essential to everyday function.
- Measures of basic activities of daily living (such as dressing, toileting, or feeding oneself), because the more limited cognitive requirements of such activities provide only a narrow view of basic aspects of cognition, but not higher-level functions such as executive and strategic processes.
- Measures single aspect of cognition only, such as executive function, because we aimed to address a broader range of cognitive skills.
- Naturalistic task.
- Measure for children.

We searched systematic reviews for measures which met our inclusion and exclusion criteria. In Stage 2 the full names of the measures found in Stage 1 were searched for in PubMed by TP and CR using a search filter developed to find studies on measurement properties to gain more detailed information.^32^ Relevant articles were imported into EndNote X9 for screening.

**Figure 1.**
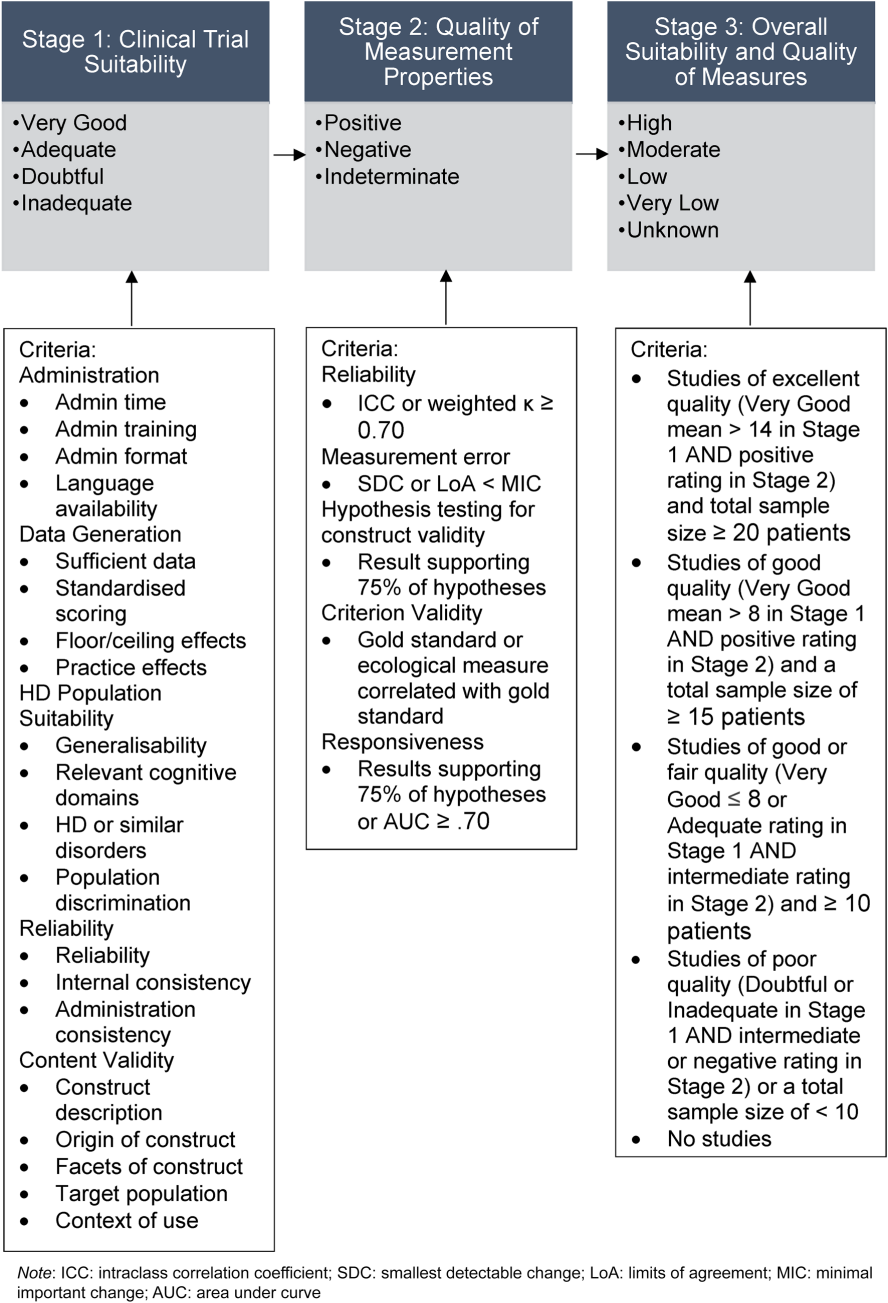
Flow chart of the three stages of measurement rating.

Measures of suitability and quality were assessed in three stages by TP and CR. In Stage 1 we assessed measure suitability for clinical trials using criteria which were developed based on feasibility and acceptability criteria for assessing cognitive outcomes in HD for a clinical trial context,^30^ and the Consensus-based Standards for the selection of health Measurement INstruments (COSMIN) content validity checklist.^31^ To refine the criteria and generate a rating system, we invited experts on clinical studies in HD for feedback on the importance and relevance of each criterion. To refine the criteria and generate a rating system, we invited experts on clinical studies in HD for feedback on the importance and relevance of each criterion. Experts were invited to provide input on the criteria based on their expertise in clinical trials or clinical outcomes in HD. Interested experts then self-selected for the study. Our final expert panel consisted of: Glenn Stebbins, a clinical neuropsychologist and professor in the Department of Neurological Sciences at Rush University Medical Centre with extensive experience in the development and validation of clinical outcome assessments for movement disorders, advanced statistical techniques, and clinimetrics; Mark Gordon, a neurologist, movement disorder subspecialist, Senior Director of Clinical Development and Neuroscience at Teva Pharmaceuticals, and clinical lead for trials in HD; Jennifer Petrillo Billet, a Senior Director at Sage Therapeutics with extensive experience in the development and validation of outcome measures; Jason Johannesen, a clinical neuropsychologists and Senior Principal Scientist at Sage Therapeutics with experience in HD clinical trial design, endpoint selection, and regulatory strategy; Rebecca Fuller, a Senior Director of Clinical Outcomes at CHDI Foundation with experience in clinical outcome development for HD trials; and Beth Borowsky, an Executive Director and Senior Global Program Clinical head at Novartis with experience in clinical development programs in HD. After sending an initial draft of the criteria, the experts rated the importance of each criterion on a scale of zero to 100% and provided comments (See Supplemental Material Appendix B). From this input, we agreed on five general categories of clinical trial suitability criteria, which were as follows: administration, data generation, HD population suitability, reliability, and content validity. Although reliability and content validity are also assessed in Stage 2 as part of the quality of measurement properties, in Stage 1 we focused on aspects of each that may be more relevant to clinical trial suitability (for example, the reliability of the administration procedures or whether measures were measuring multiple aspects of function). We used a total of 20 criteria across the five categories, as shown in Figure 1, each with specific guides for ratings of Very Good, Adequate, Doubtful, or Inadequate (see Supplemental Material Appendix C, Table C1). Of the 20 criteria, seven were identified as being desirable for use as evidence of the clinical meaningfulness of cognitive measures in HD clinical trials by the expert panel, including time efficiency, generation of data that are relevant to the cognitive aspects of HD, and the five criteria used to evaluate content validity. In Stage 2 we assessed the quality of measurement properties for each performance-based functional capacity measure according to COSMIN assessment criteria including reliability, measurement error, hypothesis testing for construct validity, criterion validity and responsiveness^33^ (see Supplemental Material Appendix C, Table C2). Overall scores of positive, negative, or indeterminate were assigned to each measure based on the combined majority rating for all measurement properties. The expert panel identified responsiveness as a desirable criterion for a functional capacity measure to provide evidence for the clinical meaningfulness of change on cognitive measures in HD.^31^

Lastly, in Stage 3 we combined the ratings from Stage 1 and Stage 2 to create overall ratings for each performance-based functional capacity measure (see Supplemental Material Appendix C, Table C3), ranging from Very Low, Low, Moderate or High according to Grading of Recommendations Assessment Development and Evaluations (GRADE).^34^ GRADE is a framework for synthesizing evidence based on sample size and quality of study methods to provide an overall rating.^34^ The overall ratings correspond to the level of confidence in the conclusions drawn or recommendations made based on the quality of the available research. For example, Very Low ratings suggest that the conclusions drawn from the evidence is uncertain, whereas Low and Moderate ratings suggest that further research is very likely and likely, respectively, to have an impact on confidence in the conclusions drawn from the evidence. High ratings are reserved only when future research is unlikely to impact or change confidence in the conclusions drawn. We followed COSMIN criteria which recommends that for outcome measures to be considered High quality, they should have studies with a total sample size of greater than 100 participants.^34^ We acknowledged that such a large sample size can be difficult to obtain in studies with neurodegenerative participants due to the effort required and, in some cases like HD, the rarity of the condition. We nevertheless chose to follow COSMIN criteria to identify studies with sufficient power to evaluate clinimetric properties. Figure 1 shows the three-stage process for rating measures.

We extracted data into a table (Table 2) listing authors, the measure name, time to administer, description of the measure, materials, participants, and clinical trial suitability and measurement properties quality scores. To analyze the data, TP wrote a narrative synthesis based on the data extraction table under four sections: search results, clinical trial suitability, measurement properties quality, and suitability to support clinical meaningfulness of cognitive measures.

**Table 2.** Measure characteristics and clinical trial suitability and measurement properties quality ratings.

Author/s	Measure	Description	Materials	Time in minutes	Participants	Overall Suitability/Quality	Stage 1	Stage 2
Goverover and DeLuca^35^ Goverover et al.^36^ Goverover and DeLuca^37^	Actual Reality™ (AR)	book airline ticket and purchase cookies and pizza online	computer, paper, pen, calendar, credit card	20	multiple sclerosis, traumatic brain injury, HC	Moderate	VG (8.67)	+ ICC .50 to .99
Scharaga and Holtzer^38^	Brief Everyday Activities Measure (BEAM)	choose breakfast items from menu and calculate cost of items and place total on desk, manage medications	menu, medication bottle, one-week pill box, wallet, money	5	healthy older adult community dwellers	Very Low	A	? ICC .65
Tanguay et al.^39^ Kosowicz and MacPherson^40^ Rose et al.^41^ Craik and Bialystok^42^	Computerized Breakfast Task/Prop-based Breakfast Task	virtually or with props cook five breakfast items while simultaneously setting table	computer with touch-screen monitor, prop-based version requires cardboard food items, timer, disposable plates and cutlery	10	acquired brain injury, HC, healthy adults and older adults	Very Low	VG (8.25)	?
Farrell et al.^29^ Hines & Bundy^43^ Johnston et al.^44^ Kay et al.^45^ Cheal et al.^46^	DriveSafe DriveAware (DSDA)	computerized driving test with three subtests: DriveSafe (visual scanning and anticipation of vehicle and pedestrian movements), DriveAware (self-awareness of driving ability) and intersection rules test	computer tablet	15	HD presymptomatic and symptomatic, stroke, Parkinson's disease, Guilan Barre syndrome, acquired brain injury, spinal injury, schizophrenia, dementia	Moderate	VG (9.80)	?
Edelberg et al.^47^ Edelberg et al.^48^	Drug Regimen Unassisted Grading Scale (DRUGS)	four tasks: identify appropriate medications, access appropriate containers, dispense correct dosage, time dosage correctly	own medications, medical record, sheet of grid paper with titles: time, meal and medications	5–7	older adults	Very Low	I	?
Book et al.^49^ Luttenberger et al.^50^ Schmiedeberg-Sohn et al.^51^ Sulzer et al.^52^	Erlangen Test of Activities of Daily Living in Persons with Mild Dementia or Mild Cognitive Impairment (ETAM)	six subtests in following order: preparing medication, making a cup of tea, evaluating traffic situations, reading and changing time on alarm clock, handling finances, making phone call	medication jars/blister pack, pills, electric kettle, bottled water, cup, tea bags, traffic situation pictures, alarm clock, shopping items & list, coins, phone, pen, paper	15–35	Parkinson's disease, mild cognitive impairment, mild dementia, moderate dementia, HC	Moderate	VG (10.75)	? ICC .96 AUC .83
Júlio et al.^14^	Eco Kitchen	three blocks that increase in difficulty: collect items in certain order, turn off the stove when clock turns red, etc.	computer	20	HD presymptomatic and symptomatic, HC	Low	VG (13)	?
Gerstenecker et al.^53^ Gerstenecker et al.^54^	Financial Capacity Instrument-Short Form (FCI-SF)	37 items assessing financial skills	testing sheet (cheque book, bank statement)	15	cognitively normal older adults, cognitively impaired adults, healthy participants	Low	A	?
Marshall et al.^55^ Marshall et al.^56^ Marshall et al.^57^	The Harvard Automated Phone Task (APT)	use phone to refill a prescription, select a physician and make a bank transfer and payment	phone with interactive voice response system	10	mild cognitive impairment, Alzheimer's disease, healthy adults and older adults	Moderate	VG (12.67)	? ICC .79
Carlson et al.^58^	Hopkins Medication Schedule	fill in schedule and pill box for hypothetical prescription	testing sheet, fake medication, pill box	12–15	healthy older adult women	Very Low	I	?
Hartman-Maeir et al.^59^ Harper et al.^60^	Kettle Test (KT)	make a hot beverage for self and therapist	kettle, cup, tea, additional kitchen utensils	5–20	stroke patients, various cognitive dysfunction, HC	Very Low	I	?
Hallowell et al.^61^ Margolis et al.^62^ Elliott and Fiszdon^63^ Bengoetxea et al.^64^ Pirogovsky et al.^65^ Patterson et al.^66^	Medication Management Ability Assessment (MMAA)	complete pill taking regimen	Pills	15	schizophrenia, Parkinson's disease, older adults, mild cognitive impairment, dementia, HC	Very Low	I	? ICC .96 AUC .76 – .96
Al-Heizan et al.^67^ Al-Heizan et al.^68^ Edwards et al.^69^	The Menu Task	select items from hospital menu	hospital menu	5	community dwelling older adults, adults hospitalized for orthopedic surgery	Low	A	? AUC .78
Beyle et al.^70^ Glonnegger et al.^71^	Multiple Object Test (MOT)	five routine tasks: make coffee, light candle, open padlock, drink water, prepare letter	kettle, cup, coffee, candle, matches, candle stand, padlock, key, glass, bottle of water, letter, envelope, stamp	15	Parkinson's disease-mild cognitive impairment, Parkinson's disease dementia, HC	Moderate	VG (9.5)	? AUC .85
Cornelis et al.^72^ Seligman et al. ^73^	Naturalistic Action Test (NAT)	three tasks: prepare toast, wrap a gift, prepare a schoolbag in lab (designed to assess cognitive abilities so examiner provides assistance with motor tasks)	bread, jelly, butter, knife, toaster, gift, gift-wrap, schoolbag, lunchbox	15	older adults, HC, mild cognitive impairment, Alzheimer's disease	Low	A	? AUC 0.81–1.00
Schmitter-Edgecimbe et al.^74^ Suchy et al.^75^ Chilton & Schmitter-Edgecombe^76^	The Night-Out Task (NOT)	eight subtasks: recipe, exit, movie, phone, snack, change, travel bag, tea	Recipe, ingredients, bag, phone, snacks, coins, tea, coffee, thermos, sheet of instructions, tablet for examiner scoring only	20	older adults, mild cognitive impairment, HC	Very Low	VG (8.33)	? ICC 0.80 – .99
*Hsieh^77^	Performance-based Instrumental Activities of Daily Living (instrumental activities of daily living)	four tasks: make change, phone use, meal preparation, medication scheduling (Hopkins medication schedule)	money, phone book page, phone, instant oatmeal, microwave, spoon, bowl, medication schedule, fake medication, pill box	20–25	older adult women, mild cognitive impairment	Low	VG (8)	? ICC .98 AUC .82
Yantz et al.^78^	Rabideau Kitchen Evaluation—Revised (RKE-Revised)	two tasks: prepare sandwich and hot beverage	containers with tea/food, stove, cup, teaspoon, knife, cutting board	15	stroke patients	Low	VG (8)	?
Vallejo et al.^79^	Serious Game	virtual cooking scenario: cooking pasta	computer tablet	25	healthy older adults	Very Low	A	?
Martinez-Pernia et al.^80^	Screen-Based Simulated Cup Of Tea (SBS-COT)	virtually make a cup of tea	touch screen computer	?	traumatic brain injury	Very Low	A	?
Jang et al.^12^ Reppermund et al.^81^	The Sydney Test of Activities of Daily Living in Memory Disorders (STAM)	nine tasks: make a phone call, put on shirt, pay a bill by cheque, prepare cheque for mailing, set an alarm, manage medications, shop for items for a recipe, calculate cost, recall activities	phone, phone book, shirt, cheque book, bill, stamp, envelope, alarm clock, medication dispenser, medication bottles, recipe, money, grocery poster, purse, testing sheet	20	community dwelling elderly, mild cognitive impairment, dementia, HC	Moderate	VG (8.5)	? AUC .72 – .95
Chen et al.^82^ Chen et al.^83^	Taiwan Performance-based Instrumental Activities of Daily Living (TPIADL)	five tasks: name food ingredients, find telephone numbers, counting coins, shop for items, medication management	plastic food kit, grocery items, coins, telephone book, medicine bottle, pictorial instructions	10	cognitively impaired elderly Taiwanese; vascular cognitive impairment, mild cognitive impairment, HC	Low	VG (8)	? AUC .89 – .90
Charvet et al.^84^	The Test of Everyday Cognitive Ability (TECA)	domains of communication, finance, nutrition, shopping and medicine: find telephone number, make change, read soup label, find food items, read medicine bottle, buy food items, review shopping list	brand free grocery props, mock pill bottles, money, shopping list	25	multiple sclerosis, HC	Low	VG (8)	?
Cullum et al.^85^ Crawford et al.^86^ Gonzalez et al.^87^ Lowe et al.^88^ Lowe & Linck^89^ Roye et al.^90^	The Texas Functional Living Scale (TFLS), also Test of Everyday Functional Abilities (TEFA)	make change, tell time, dial phone number, use calendar, medication management	money, clock, phone, phone book, calendar, medication	15–20	Alzheimer's disease, HC, older adult veterans with dementia, mild cognitive impairment, major depressive disorder, posttraumatic stress disorder, age-related neurocognitive disorders, and older adult outpatients	Low	VG (7.17)	?
Owsley et al.^91^	Timed Instrumental Activities of Daily Living (TIADL)	use telephone, nutrition, financial abilities, shopping, medication management	real everyday objects and kit including small shelf for bottles, food cans, telephone directory, coins	15	healthy older adults	Very Low	A	?
Goldberg et al.^92^ Gomar et al.^93^ Moore et al.^94^ Vella et al.^95^ Sheppard et al.^96^ Sumiyoshi et al.^97^ Mausbach et al.^98^ Mausbach et al.^99^ Mausbach et al.^100^ Leifker et al.^101^	The University of California, San Diego (UCSD), Performance-based Skills Assessment Brief (Holden-B)	three subtests: comprehension and planning, communication, and finance, e.g., understand written material on recreational outing/plan activities, role play phone calls, make change, fill out cheque	written material, phone, phone book, money, cheque, utility bill	10–15	mild cognitive impairment, Alzheimer's disease, schizophrenia, HIV/AIDS acquired neurological disorder, healthy adults, bipolar, HC	Moderate	VG (10.6)	? AUC .70 – .84
Moore et al.^102^	The University of California, San Diego (UCSD), Computerized Performance-based Skills Assessment (C-UPSA)	four computerized subtests: planning recreational activities, finance, communication and transportation, e.g., plan recreational outing, role play phone calls, make change, navigate bus route	computer	20	schizophrenia	Very Low	VG (14)	? AUC .88
Moore et al.^103^	The University of California, San Diego (UCSD), Mobile Performance-based Skills Assessment (UPSA-M) Brief	optional four (planning recreational activities, finance, communication and transportation), or two computerized subtests (finances and communication)	computer tablet, stylus	10 (brief) 25 (full)	schizophrenia	Very Low	VG (14)	? AUC .80 – .87
Czaja et al.^104^ Czaja et al.^105^	University of Miami Computer-Based Functional Assessment Battery (UMCFAB); also referred to as Automatic Teller Machine	computerized money management by using an Automatic Teller Machine (ATM) machine, prescription refill task	computer (touchscreen optional)	15	schizophrenia, healthy older adults, mild cognitive impairment	Moderate	VG (13.5)	?
Sorita et al.^106^ Aubin et al.^107^	Virtual Action Planning Supermarket (VAP-S)	supermarket simulation of performing shopping task	computer (projector optional)	4–38	acquired brain injury, schizophrenia	Low	A	?
Allain et al.^108^ Besnard et al.^109^	The Virtual Kitchen; also called Nonimmersive Virtual Coffee Task (NI-VCT)	virtually prepare coffee	computer	20	Alzheimer's disease, older adults	Low	VG (10.5)	?
Giovannetti et al.^110^ Holmqvist et al.^111^	Virtual Kitchen Challenge (VKC)	virtually prepare breakfast and lunch modelled on NAT	computer (touchscreen optional)	20	healthy young and older adults, mild cognitive impairment	Very Low	VG (10.5)	?
Atkins et al.^112^ Atkins et al.^113^ Harvey et al.^114^ Keefe et al.^115^ Lindenmayer et al.^116^ Ruse et al.^117^ Ruse et al.^118^ Turner et al.^119^ Ventura et al.^120^	The Virtual Reality Functional Capacity Assessment Tool (VRFCAT)	virtually navigate a kitchen, catch a bus to the grocery store, find/purchase groceries, return home on bus	computer	15–35	older adults, schizophrenia, Parkinson's disease, mild cognitive impairment	Moderate	VG (10.33)	? ICC .61 – .81

*Note: *A: Adequate; HC: healthy control; HD: Huntington's disease; HIV/AIDS: human immunodeficiency viruses/acquired immunodeficiency syndrome; ICC: intraclass correlation coefficient; I: Inadequate; PD: Parkinson's disease; VG: Very Good.

? = indeterminate; - = negative; + = positive.

*Dissertation.

## Results

### Search results

**Figure 2.**
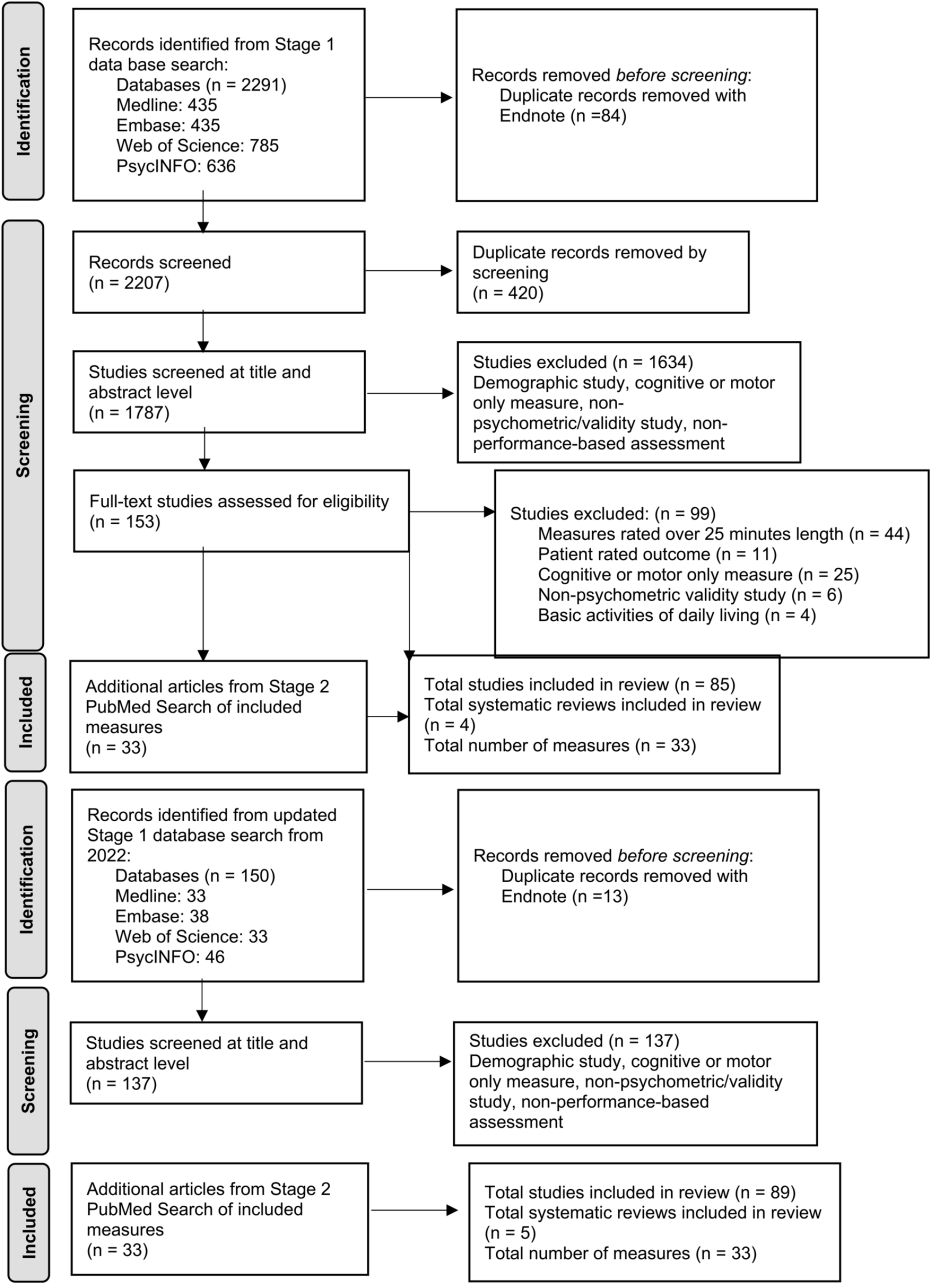
PRISMA flow chart of article and measure selection.

Five systematic reviews of performance-based functional capacity measures were found in the Stage 1 search from which some measures were included.^121–125^ One of these systematic reviews focused solely on one performance-based measure of function, DriveSafe DriveAware.^125^ Figure 2 shows the process of the Stage 1 and Stage 2 database search. We extracted the data from a total of 89 articles on 33 different measures into a data extraction table containing each measure's description, materials, administration time, participant groups, and clinical trial suitability and measurement properties quality ratings (see Table 2). Overall, two measures constituted the most articles, the Brief University of California, San Diego (UCSD), Performance-based Skills Assessment (UPSA-B) and the Virtual Reality Functional Capacity Assessment Tool (VRFCAT), with 10 and nine articles respectively. Thirteen of the measures were in a computerized format and 21 required props such as grocery items and medication bottles. Two of the measures, the Breakfast Task and UCSD UPSA-B, had articles on administration in both computerized and prop-based form. Sixteen of the measures were rated Very Good for the facets of the construct measured, meaning their assessment of function was multi-faceted, such as the VRFCAT and the UPSA-B. The remaining 17 measures only measured one or very few facets of function; for example, the Medications Management Ability Assessment (MMAA), which only examined function with regard to medication management. Only two measures, DriveSafe DriveAware and EcoKitchen, had been tested in a HD population.

### Clinical trial suitability assessment

In Stage 1, ‘clinical trial suitability assessment overall’, we rated 21 measures as Very Good, eight as Adequate and four as Inadequate. For measures with Very Good ratings, a mean Very Good rating (see Table 2) was calculated by combining the number of Very Good ratings and dividing it by the total number of articles for that measure. Measures with the highest ratings were the UCSD UPSA computer and mobile versions with a mean of 14 Very Good ratings each across all five categories of the clinical trial suitability criteria. All measures had articles that were rated as having Adequate to Very Good levels of sufficient data on functional capacity, according to the measures producing multiple data points or having items with sufficient clinical meaningfulness. Only six of the 33 measures evaluated floor and ceiling effects and found none to be present.

### Measurement properties quality assessment

In Stage 2, ‘measurement properties quality assessment’, only one measure, Actual Reality, received a positive rating overall. This rating was based on multiple articles finding acceptable test-retest and inter-rater reliability as indicated by intraclass correlation coefficients (ICC) > 0.70, and by demonstrating sufficient construct validity. As most studies made hypotheses based on assumptions or expectations of the underlying construct of the measure (such as that it will relate to other similar measures, or that it will be able to distinguish between better or worse function), construct validity was indicated when 75% of the study hypotheses were supported. The remaining 32 measures received an indeterminate rating due to lack of information regarding their measurement properties. Only eight out of 33 measures had documented ICC, with most articles using Pearson correlations, which are not considered as accurate to determine test-retest and interrater reliability.^126^

### Overall clinical trial suitability and measurement properties quality ratings

In Stage 3, we combined the ratings from Stage 1 and Stage 2 for each of the measures to receive an overall rating of either Very Low, Low, Moderate, or High. Nine measures received Moderate ratings based on the publications on these measures being rated as Very Good (mean rating > 8) for clinical trial suitability, a positive (or indeterminate, meaning lacking information on measurement properties) score for the quality of the measurement properties, and neurodegenerative population sample sizes ≥50. Moderate ratings mean that while the available evidence suggests it may be suitable for use in HD clinical trials, confidence in their suitability is likely to be impacted by future research. This distinction is especially important given almost all measures received an indeterminate rating for the quality of their measurement properties. Eleven measures received Low ratings based on having Very Good (mean rating < 8) or Adequate clinical trial suitability, an indeterminate rating for measurement property quality, and neurodegenerative population sample sizes ≥30. Thirteen measures received Very Low ratings based on Inadequate clinical trial suitability, indeterminate or negative measurement property quality, and neurodegenerative population samples sizes < 30. None of the measures received High ratings, which were reserved only when future research was unlikely to change confidence in the suitability in measures. This was defined as receiving Very Good clinical trial suitability ratings (mean rating > 14), positive measurement property quality, and neurodegenerative population sample sizes ≥ 100 across multiple studies. Of the measures that received an overall Moderate rating, several included validation studies with over 100 participants, including the UCSD UPSA-B, DriveSafe DriveAware, and the VRFCAT. Measures that have conducted validation with large participant groups can more robustly determine quality of psychometric properties.

### Suitability to support clinical meaningfulness of cognitive measures

Our expert panel identified several desirable characteristics of the performance-based measures of functional capacity identified in our literature search for use as evidence of the clinical meaningfulness of cognitive measures. These characteristics included time efficiency, the generation of data relevant to the cognitive aspects of HD, and content and construct validity. Although most measures had sufficiently brief administration times in healthy controls, which we defined as under 25 min, seven of the measures had longer administration times of up to around 35 min (e.g., VRFCAT, ETAM). All measures included studies that were rated as Adequate to Very Good at producing data with relevance to cognitive domains affected by HD, such as executive function. Most of the articles omitted information on floor or ceiling effects. Most measures, however, for which range limitations were considered, revealed at least one publication where floor or ceiling effects had been considered and were absent. These included the Harvard Automated Phone Task, the Texas Functional Living Scale, the Timed Instrumental Activities of Daily Living, the University of Miami Computer-Based Functional Assessment Battery and the UCSD UPSA-B. Of note, however, evidence for the UCSD UPSA-B was inconsistent, with some publications indicting ceiling effects.^95,96^

Construct validity is the degree to which a measure is capturing the underlying constructs that it claims to be measuring whereas content validity is the degree to which a measure is capturing all facets of a construct. In this regard, we rated 30 of the 33 measures as having Adequate to Very Good content validity based on providing a clear description of function as a construct, defining the target population, and described the context of use such as the stage of the disease for which the measure was designed. The only four that did not obtain these high ratings were Actual Reality, Computerized Breakfast Task, and the Virtual Action Planning Supermarket. Measures that were multi-faceted and assessed multiple aspects of function, such as the VRFCAT, measured the construct of function more comprehensively than single-facet measures, such as the Financial Capacity Instrument-Short Form, suggesting that these measures had greater content validity. In addition to content validity, we also considered responsiveness as relevant to the construct validity of a measure. Responsiveness can be used to demonstrate construct validity because it indicates whether a measure is sensitive to changes in the underlying construct: function. About half of the articles included evidence of responsiveness, demonstrating measure sensitivity in discriminating participants with disorders from healthy controls. The measures with particularly high responsiveness were all versions of the UCSD UPSA and the Sydney Test of Activities of Daily Living in Memory Disorders.

## Discussion

Performance-based outcome measures of functional capacity have important potential for use in HD clinical trials to assess the benefit of treatments, and to establish the clinical meaningfulness of cognitive outcome measures. The primary aim of our review was to identify performance-based functional capacity measures and assess their potential suitability for use in HD clinical trials. We found 33 performance-based functional capacity measures with a range of suitability from Very Low to Moderate based on administration factors, characteristics of the data they generate, their suitability for the HD population, as well as reliability, content validity and measurement properties. Our review uncovered several functional performance-based measures that, with further development and testing, could be suitable for use in HD clinical trials. These include UCSD UPSA-B ,^92–97,99–101^ as well as the VRFCAT.^113–120^ The UPSA-B and VRFCAT are also the most studied and had the most data available according to our review. Importantly, the UCSD UPSA computerized and mobile versions received the highest ratings for their Clinical Trial Suitability but had an overall Very Low rating due to limited evaluation of psychometric properties and small sample sizes. Further evaluation is needed with larger samples sizes of relevant participant groups to support their suitability for HD clinical trials.

Another goal of our review was to evaluate the measures based on desirable characteristics that increase their suitability to be used as coprimary outcome measures with cognitive measures in HD clinical trials. Desirable characteristics we considered included time efficiency, their generation of data relevant to cognition, and high construct validity. Most of the included measures are likely to be suitable for use as a coprimary measure to cognitive measures based on time efficacy, using a 25-min duration as a target, fitting within the time constraints of a typical clinical trial with multiple outcome measures. Of note, the duration of the VRFCAT is up to 35 min in people with some disorders, which potentially limits its suitability in clinical trials. One study did use an abbreviated version of the VRFCAT which took approximately 15 min.^119^ Most of the performance-based functional capacity measures we found also produced sufficient data with relevance to aspects of cognition affected in HD.

Guidance from the U.S. Food and Drug Administration (FDA) emphasizes the importance of using outcome measures that can feasibly observe changes within the context and duration of a clinical trial.^127^ As such, considering ceiling effects (i.e., consistent performances at maximum scores in a studied sample) is vital. Functional capacity measures not subject to ceiling effects unsurprisingly yielded a greater amount of useful data, with better sensitivity to change in a person's condition, including their cognition, and are therefore more suitable as coprimary outcomes to cognitive measures. A functional capacity measure that performed well in terms of having an unrestricted range at the better performing end of the scale was the University of Miami Computer-based Functional Assessment Battery, which increases its value as a potential coprimary outcome.

For suitability as a co-primary measure in a clinical trial, we also required that a performance-based functional capacity measure demonstrate construct and content validity to substantiate their relevance to real-life function across multiple facets.^31^ Regulatory guidance from the FDA and European Medicines Agency (EMA) have suggested that a broad range of effects on measures of cognition and on everyday function would be persuasive to support clinically meaningful change in other neurodegenerative disorders, such as Alzheimer's disease.^23,127^ As such, we considered multi-faceted measures which assessed multiple aspects of function and underlying cognition as more suitable candidates for coprimary outcome measures. Single-facet functional capacity measures, such as DriveSafe DriveAware and EcoKitchen^14,29,43–46,125^ were more limited in their coverage of the construct of function in that they used only a single task with limited generalizability to the broader concept of function. Measures with a broad range of tasks, such as the UCSD UPSA and VRFCAT as more suitable coprimary outcome measures to assess cognition.

### Strengths and limitations of the review

We completed a comprehensive two-stage literature search, which allowed for the identification of a wide range of measures. We used a small expert panel to assess the criteria for assessing clinical trial suitability of measures and measurement properties instead of a widely known or accepted framework, which is a limitation of our review. A larger-scale Delphi study with feedback from a large group of experts, such as was conducted to develop the COSMIN criteria,^33,128^ was not feasible for us due to time and funding limits. Given the lack of previous analyses and the high level of the experts we worked with, we believe this work is valuable for the field and sufficient to inform the next stages of consideration for the use of functional performance-based measures for HD clinical trials. Our rating scheme was adapted from previous research using the well-established GRADE and COSMIN guidelines, creating a comprehensive and rigorous appraisal system. Due to some of the limitations inherent in neurodegenerative diseases, such as rarity of diseases and challenging recruitment, many studies included in this review were underpowered to robustly evaluate the psychometric properties of a measure. Some measures may have high potential suitability for use in clinical trials for HD but have received low ratings overall due to sample size. More research is needed with large, robust sample sizes to fully evaluate the psychometric properties of candidate measures before they can be conclusively recommended for use in clinical trials. Finally, few studies identified by our review analyzed psychometric properties of performance-based measures, with only one measure out of the 33 obtaining a positive rating for the quality of measurement properties. All other measures received an indeterminate rating due to lack of information on measurement properties, including those that have been used in HD populations. Without sufficient psychometric evaluation, the true suitability of measures for use in clinical trials is difficult to determine. Increased attention to psychometric properties will be essential to advance this area of clinical outcome measurement.

### Implications and significance

Currently limited consensus exists on which measures of functional capacity to use in HD clinical trials. Existing rating scales are insensitive to early changes in HD and require further evaluation of psychometric properties.^19^ For example, the most widely used measure, the clinician-rated United HD Rating Scale (UHDRS) Total Functional Capacity (TFC) scale has limitations including reliance on participant insight for information and reduced sensitivity to mild impairment.^6,8–10,16^ As performance-based measures are based on direct observation of participant function, they are not reliant on participant insight or care partner biases, making them more reliable for use as clinical trial outcome measures. Additionally, measures sensitive to symptom progression are needed in HD clinical trials to track declines or improvements in function.^30^ Several of the measures we reviewed demonstrated no or negligible ceiling effects, including the UPSA-B and the University of Miami Computer-based Functional Assessment Battery, indicating that they are likely to be sensitive to small changes. The included measures also demonstrated their ability to discriminate well between participants with disorders and healthy controls with high levels of responsiveness, further supporting their feasibility for use in HD clinical trials.

The FDA recommends consensus on measures used across clinical trials to allow for comparison of treatments effects across studies.^2^ Our review contributes toward identification of performance-based functional capacity measures that may be useful in HD clinical trials by evaluating evidence and making recommendations for suitable measures. Consensus on the most suitable measure of functional capacity for HD clinical trials should be based on head-to-head comparisons of the best options, which will then lead to more effective endpoint strategies. Such an approach would also help to support the case to regulators of the clinical meaningfulness of potential treatment effects. Once these better-informed trial endpoints are established, the approval of novel treatments for HD can become more efficient and consistent, in turn accelerating the access of people with HD to beneficial treatments. To ensure treatment benefit is accurately assessed, meaningful reflection of everyday function by measures in clinical trials is also advised by the FDA.^129,130^ The suitable performance-based functional capacity measures that we identified in this review have the potential ability to meaningfully reflect function, and therefore to support claims of clinical meaningfulness of cognitive measures.

### Future directions

Future studies of performance-based functional capacity measures should assess measurement properties methodically and comprehensively to allow for easier comparison and compilation of results from multiple studies on the same measure. Research is needed regarding the construct validity of performance-based functional capacity measures, and such studies need to test clearly defined hypotheses presented with clear indications for how they will be tested. The identification of suitable performance-based functional capacity measures is also hampered by the absence of an explicit best practice framework, or a gold standard measure of function, with which other measures can be compared. To work towards this framework, an in-depth examination of correlations between performance-based functional capacity measures and self-report, informant, and clinician-rated measures of function is warranted. Performance-based functional capacity measures could also be evaluated in both computerized and prop-based forms to determine impact of administration method on participants’ performance. Some measures such as the UCSD UPSA in computerized and mobile formats show considerable promise for their clinical trial suitability; however, they have undergone limited psychometric evaluation and were only tested in small samples. Future research should include these measures in larger-scale studies to further support their utility in clinical trials. The most suitable measures found in our review, including the UCSD UPSA and VRFCAT, require testing in participants with HD before use in clinical trials. Including these measures in larger longitudinal studies such as Enroll-HD would be ideal to evaluate psychometric properties such as test-retest reliability with a large cohort of HD participants. Finally, although suitable single-facet measure DriveSafe DriveAware have been tested in participants with HD, it is important to test multi-facet measures that may assess the construct of function more completely. Such studies should methodically evaluate the reliability, validity and clinical trial suitability of performance-based functional capacity measures.

## Conclusion

In summary, multiple performance-based functional capacity measures are suitable for use in HD clinical trials, including as an adjunct to cognitive measures to demonstrate their clinical meaningfulness. A consensus on which functional capacity measures to use in HD clinical trials needs to be established through specific testing in HD and further investigation of their measurement properties. Aligning on functional capacity measures can facilitate discussions with regulatory authorities and potentially lead to approval of novel treatments for HD patients.

## Supplemental Material

sj-docx-1-hun-10.1177_18796397251330846 - Supplemental material for A systematic review of performance-based functional capacity measures for use in Huntington’s disease and evaluation of their suitability for clinical trials Supplemental material, sj-docx-1-hun-10.1177_18796397251330846 for A systematic review of performance-based functional capacity measures for use in Huntington’s disease and evaluation of their suitability for clinical trials by Tayela M Prichard, Cali M Roiboit, Meg E Rankin, Yifat Glikmann-Johnston, Mark F Gordon and Julie C Stout in Journal of Huntington's Disease
