# Supplementary material for: A systematic review of performance-based functional capacity measures for use in Huntington’s disease and evaluation of their suitability for clinical trials
Source: J Huntingtons Dis. 2025 Apr 3;14(2):113–31. doi: 10.1177/18796397251330846 (PMC12231780; doi:10.1177/18796397251330846)
Supplement: sj-docx-1-hun-10.1177_18796397251330846 - Supplemental material for A systematic review of performance-based functional capacity measures for use in Huntington’s disease and evaluation of their suitability for clinical trials [file sj-docx-1-hun-10.1177_18796397251330846.docx]

**Supplemental Material**

**A systematic review of performance-based functional capacity measures for use in Huntington’s disease and evaluation of their suitability for clinical trials**

**Appendix A**

**Table A1.** Literature Search Strategy Ovid Embase MeSH Terms and Text-words

| MeSH | daily life activity/ AND psychological tests/ OR neuropsychological assessment/ AND Alzheimer Disease/ OR exp Huntington chorea/ OR Parkinson disease/ OR *Cognitive defect / di [Diagnosis] AND exp Psychometrics/ |
| --- | --- |
| Text-words | activit* daily li* OR function NOT executive function* AND measure OR assessment OR test OR tool AND Huntington* OR Westphal variant OR Alzheimer* OR Parkinson* AND validity AND performance AND cognit* |

**Table A2.** Literature Search Strategy Ovid PsycINFO MeSH Terms and Text-words.

| MeSH | activities of daily living/ AND Psychological Assessment/ OR Neuropsychological Assessment/ AND Alzheimer disease/ OR Huntingtons Disease/ OR Parkinson disease/ OR Cognitive Impairment AND exp Psychometrics/ |
| --- | --- |
| Text-words | function* OR activit* daily li*AND measure OR assessment OR test OR tool AND Huntington* OR Westphal variant OR Alzheimer* OR Parkinson* AND validity AND performance |

**Table A3.** Literature Search Strategy Web of Science Core Collection search terms.

| Text-words | Function AND measure OR assessment OR test AND Huntington* OR Parkinson* AND performance AND validity OR psychometrics |
| --- | --- |

**Appendix B**

Example of expert panel consensus materials from Stage 1.

**Performance-based Functional Capacity Measure**

HD Clinical Trial Suitability Criteria and Quality Assessment

Please write any comments and rate each criterion’s importance from 1 (not important) to 100 (highly important) in the % column.

**Stage 1: Clinical trial suitability criteria**

Each measure’s overall rating is “Very good”, “Adequate”, “Doubtful” or “Inadequate” based on the rating with highest frequency. If ratings are of equal frequency the lower rating is selected.

| **Criterion** | **Very good** | **Adequate** | **Doubtful** | **Inadequate** | **Comments** | **%** |
| --- | --- | --- | --- | --- | --- | --- |
| **Administration*** Would also add – are additional languages available | | | | | | |
| Is the measure time efficient? | ≤ 20 min | ≤ 25 min | ≤ 50 min | > 50 min | ok  If the perfect measure takes too long people aren’t going to use it | 80  70  80  90 |
| Is the time required for training raters acceptable? | ≤ 30 min | ≤ 40 min | ≤ 50 min | > 50 min or not listed | ok  We need to be able to train adequately | 30  50  50  85 |
| Is the format of the measure easily administrable? | Mobile or computer-based or no physical materials required | Some physical materials required | Many physical materials required | Materials required unclear | Could also add: Is a single required physical device or can it be flexible to any physical devices  Too many physical materials is a problem, but  I am not sure it has to be 100% remote to be a very good assessment | 50  80  85  90 |
| Data generation* | | | | | | |
| Does the measure produce sufficient data? | Multiple data points or items |  | Few data points or items | One data point or item | The clinical meaningfulness of the data points seems more important than the # of data points.  Might increase this to 90% since we are talking about performance-based measures. It is good to have a single composite score constructed on multiple data points, but a scale that only produces a single score would be of limited value given current understanding of endpoints  There has to be enough data collected to show some variance | 20  70  90  75  90 |
| Is the scoring method standardized to limit error and improve accuracy? | Computerized or detailed method with high accuracy |  |  | Unclear method with low accuracy | ok  I like this | 50  70  90  90 |
| HD population suitability* | | | | | | |
| Is the measure/measure scenario generalisable? | Measure generalisable to participants of varied generations and cultures |  |  | Measure not generalisable to participants of varied generations and cultures | ok  What if it can be easily adapted for multiple cultures? | 80  70  85  90 |
| Is the measure of relevant cognitive domains for the HD population? e.g., executive function, prospective memory | Multiple HD relevant cognitive domains measured | HD relevant cognitive domain measured |  | Cognitive domain not relevant to HD not measured or specified | ok  Would de-emphasize somewhat given that we tend not to see 1:1 relationships between cognitive functions defined by neuropsychological tests and performance-based metrics of function. If the performance-based measure works, would be less concerned about its relationship to an established set of cognitive domains for HD  Must test the important domains. How is it decided which domains are the most important? | 90  100  85  100 |
| Reliability | | | | | | |
| Is there evidence of any kind of reliability for the measure? | Evidence of reliability |  |  | No evidence of reliability | Does this need to be more precise?  Does any reliability evidence mean it is a very good scale, such as a single test-retest administration? However I accept that it would be hard to define good vs lame evidence of reliability. | 50  80  100  80  90 |
| Content validity** | | | | | | |
| Is a clear description provided of the construct to be measured? | Construct  clearly  described | Some description provided but not thoroughly presented |  | Construct not  clearly  described | ok  This is useful | 60  100  80  90 |
| Is the origin of the construct clear: was  a theory, conceptual framework or  disease model used or clear rationale  provided to define the construct to be  measured? | Origin of the  construct clear | Some description provided but not thoroughly presented | Origin of the  construct not  clear |  | ok  Might de-emphasize this a little given that theoretical and conceptual frameworks may guide development, but empirical basis is most critical to test performance  This is useful | 50  100  75  90 |
| Is a clear description provided of the target population for which the measure  was developed? | Target  population  clearly  described | Some description provided but not thoroughly presented |  | Target  population not  clearly  described | ok  Very important. If an assessment was created for one indication, there should be a study to show it is viable for the target indication before recommending it. | 90  100  75  100 |
| Is a clear description provided of the context of use? e.g. stage of the disease, mode of administration, setting | Context of use  clearly  described |  |  | Context of use  not clearly  described | ok  Very important, especially in HD | 90  100  80  90 |

*Criteria based on Stout, Andrews & Glikmann-Johnston, 2017

**Criteria adapted from Terwee et al., 2018

**Comments**

You have validity before reliability, and as you know, the validity coefficient cannot exceed the reliability coefficient squared. I don’t think this will be important to the presentation; most folks think validity is the most important metric, but we should keep the true order of importance in mind when reviewing the scales.

We all felt internal reliability was extremely important. We are very keen to see the criteria for the psychometric data reviews, as we think responsiveness holds the key to the kingdom here.

In terms of psychometric support, the scale should demonstrate adequate discriminant validity with respect to HD staging (i.e., sensitivity to progression of functional decline)

What is missing is a questions on the effect size seen in the HD population, either cross-sectionally or longitudinally. Also, important is any known treatment effects in HD or other conditions.

Measure should be able to realistically change within time frame of the trial, e.g. sensitive

- practice effects

**Appendix C**

HD Clinical Trial Suitability and Quality of Measurement Properties Criteria

Stage 1: Clinical trial suitability

Each measure’s overall rating is “Very good”, “Adequate”, “Doubtful” or “Inadequate” based on the rating with highest frequency. If ratings are of equal frequency the lower rating is selected. Measures with Very Good ratings have a score calculated by scoring the mean number of Very Good ratings across all articles.

Table C1

***Criteria for rating clinical trial suitability***

| Criterion | Very good | Adequate | Doubtful | Inadequate |
| --- | --- | --- | --- | --- |
| **Administration*** | | | | |
| Is the measure time efficient? | ​​≤ ​20 min single administration time including participant training | ​​≤ ​25 min single administration time including participant training | ​​≤ ​50 min single administration time including participant training | > 50 min single administration time including participant training |
| Is the time and method for training administrators acceptable? | ​​≤ 3​0 min or detailed method | ​​≤ 4​0 min or adequately described method | ​​≤ 5​0 min or unclear method  or not listed |  |
| Is the format of the measure easily administrable? | Mobile or computer-based or few physical materials required | Some physical materials required | Many or very specific physical materials required | Materials required unclear |
| Are versions of the measure in additional languages available? | Versions in additional languages with appropriate translation and cultural development described | Versions or plans to develop versions in additional languages |  | No versions or no plans to develop versions in additional languages or not known |
| **Data generation*** | | | | |
| Does the measure produce sufficient data? | Multiple data points or items with sufficient clinical meaningfulness^a^ | Few data points or items with some clinical meaningfulness^a^ | Few data points or items with low clinical meaningfulness^a^ | One data point or item  with low or unclear clinical meaningfulness^a^ |
| Is the scoring method standardized to limit error and improve accuracy? | Computerized or detailed method with high accuracy | Briefly described method with moderate accuracy |  | Unclear method with low accuracy |
| Have floor and ceiling effects been evaluated? | No or negligible floor or ceiling effects |  | Minor floor or ceiling effects evidenced | Floor or ceiling effects evidenced or not evaluated |
| Have practice effects been evaluated? | Practice trials incorporated and effects have been examined | Practice trials incorporated but effects not examined or unclear |  | Practice trials not incorporated, and effects not examined |
| **HD population suitability*** | | | | |
| Is the measure/measure activity generalisable? | Measure generalisable to participants of varied generations and cultures | Measure adaptable for participants of varied generations and cultures |  | Measure not generalisable or adaptable for participants of varied generations and cultures |
| Is the measure of relevant cognitive domains for the HD population? e.g., executive function, prospective memory | HD relevant cognitive domain/s specified | Some relevance to HD cognitive domain/s |  | Cognitive domain/s not specified |
| Has the measure been used in participants with HD or similar neurodegenerative conditions? | Measure has been used in participants with HD | Measure has been used in participants with conditions similar to HD |  | Measure has not been used in participants with HD or similar conditions |
| Does the measure discriminant between participants with neurodegenerative conditions and healthy controls? | Evidence the measure discriminates between groups | Some evidence the measure discriminates between groups |  | No evidence the measure discriminates between groups |
| **Reliability** | | | | |
| Is there evidence of reliability (e.g. interrater, test-retest)? | Sufficient evidence of reliability | Some evidence of reliability |  | No evidence of reliability |
| Is there evidence of internal consistency if the measure is based on a reflective model^b^, or is the measure based on a formative model^b^? | Evidence of internal consistency, or model is based on a formative model^b^ | Some evidence of internal consistency |  | No evidence of internal consistency |
| Were administration procedures consistent across administrations and/or studies? | Detailed evidence of consistent administration procedures | Some evidence of consistent administration procedures |  | Unclear or inconsistent administration procedures |
| **Content validity**** | | | | |
| Is a clear description provided of the construct to be measured? | Construct  clearly  described | Some description provided but not thoroughly presented |  | Construct not  clearly  described |
| Is the origin of the construct clear: was  a theory, conceptual framework or  disease model used or clear rationale  provided to define the construct to be  measured? | Origin of the  construct clear | Some description provided but not thoroughly presented or rationale not completely clear | Origin of the  construct not  clear |  |
| Are multiple facets of the construct measured? | Multiple facets measured | Few facets or a singular facet measured | Facets measured unclear |  |
| Is a clear description provided of the target population for which the measure  was developed? | Target  population  clearly  described | Some description provided but not thoroughly presented |  | Target  population not  clearly  described |
| Is a clear description provided of the context of use? e.g. stage of the disease, mode of administration, setting | Context of use  clearly  described | Some description of context of use provided but not thoroughly presented |  | Context of use  not clearly  described |

*Criteria based on Stout et al.^30^

**Criteria adapted from Terwee et al.^31^

^a^ Clinical meaningfulness is defined as the measure’s ability to determine clinically important changes in a participant’s condition

^b “^A reflective model is a model in which all items are a manifestation of the same underlying construct. These items are called effect indicators and are expected to be highly correlated and interchangeable. Its counterpart is a formative model, in which the items together form a construct. These items do not need to be correlated. Therefore, internal consistency is not relevant for items that form a formative model.” p3, Mokkink et al.^131^

Stage 2: Quality of measurement properties

The measurement properties of each FC measure are rated “positive”, “negative” or “indeterminate” according to COSMIN criteria for good measurement properties (Prinsen et al., 2016), adapted by Jakobsson et al. (2019) for performance-based measures (see Table 1). For each of the five measurement properties, total rating is based on the majority positive or negative rating combined across all papers if more than one paper is available. For a measurement property that has only one paper with a positive or negative rating or all ratings are indeterminate, the rating for that property is indeterminate. If ratings are of equal frequency the lower rating is selected. The majority positive, negative or indeterminate rating for all measurement properties combined across all papers on a measure is the overall rating for each measure.

**Table C2.** Criteria for rating quality of measurement properties.

| Measurement property^a^ | Rating^b^ | Criteria for rating quality |
| --- | --- | --- |
| Reliability | + | ​​ICC or weighted κ ≥0.70 ​ |
|  | ? | ICC or weighted κ not reported |
|  | - | Criteria for “+” not met |
| Measurement error | + | SDC or LoA < MIC^c^ |
|  | ? | MIC not defined |
|  | - | Criteria for “+” not met |
| Hypothesis testing for construct validity | + | 75% of the results in accordance with the hypotheses |
|  | ? | No hypotheses defined |
|  | - | Criteria for “+” not met |
| Criterion | + | ​​Convincing argument that gold standard is ‘gold’ or valid ecological measure AND correlation with gold standard ≥0.70​ |
|  | ? | Not all information for “+” reported |
|  | - | Criteria for “+” not met |
| Responsiveness | + | ​​75% of the results in accordance with the hypotheses or AUC ≥ 0.70​ |
|  | ? | No hypotheses defined |
|  | - | Criteria for “+” not met |

^a^Based on Prinsen et al., 2016; AUC = area under the receiver operating characteristic curve; ICC = intraclass correlation coefficient; LoA = limits of agreement; MIC = minimal important change; SDC = smallest detectable change. ^b^​+ = positive rating; ? = indeterminate rating; − = negative rating. ​^c^This evidence can come from different studies.

Stage 3: Best Evidence Synthesis: Overall Suitability and Quality of Measures

Ratings from Stage 1 and Stage 2 are combined to form an overall suitability and quality rating in line with the Grading of Recommendations Assessment, Development and Evaluation (GRADE) working group.^34^

Quality is defined as:

- excellent quality: Stage 1 (very good mean ​≥​14) AND Stage 2 (positive)
- good quality: Stage 1 (very good mean >8) AND Stage 2 (positive OR indeterminate)
- fair quality: Stage 1 (very good ​≤​8 or adequate) AND Stage 2 (indeterminate)
- poor quality: Stage 1 (doubtful or inadequate) AND Stage 2 (indeterminate OR negative)

**Table C3.** Criteria for overall suitability and quality of measures

| Quality rating | Criteria* |
| --- | --- |
| High | Studies of excellent quality AND a total sample size of ≥100 patients ​ |
| Moderate | Studies/study of good quality ​AND a total sample size of ≥50 patients​ |
| Low | Studies/study of good quality OR studies ​of fair quality AND a total sample size of ≥30 patients​ |
| Very Low | Studies/study of poor quality AND/OR a total sample size of <30 patients |
| Unknown | No studies |

*Adapted from Prinsen et al.^33^
